# Evaluation of early left-sided cardiac reverse remodeling under combined therapy of sacubitril-valsartan and spironolactone compared with angiotensin-converting enzyme inhibitors and spironolactone

**DOI:** 10.3389/fcvm.2023.1103688

**Published:** 2023-04-03

**Authors:** Wioletta Sacharczuk, Rafał Dankowski, Stefan Ożegowski, Maciej Rojna, Andrzej Szyszka

**Affiliations:** Second Department of Cardiology, Poznan University of Medical Sciences, Poznan, Poland

**Keywords:** sacubitril-valsartan, spironolactone, cardiac reverse remodeling, global longitudinal strain, left ventricular ejection fraction, indexed left atrial volume, heart failure

## Abstract

We aimed to compare therapies of sacubitril/valsartan + spironolactone (S/V + S) with angiotensin-converting enzyme inhibitors + spironolactone (ACEI + S) on the left-sided cardiac reverse remodeling (L-CRR). The second objective was to analyze the usefulness of GLS and LVEF in response to therapy.

**Methods:**

78 patients (mean age 63.4 years, 20 females) with symptomatic heart failure with reduced ejection fraction were randomized to groups of equal numbers, i.e., 39 patients, and started on therapy of S/V + S or ACEI + S. Second evaluations were made after 6–8 weeks of therapy.

**Results:**

GLS changed from −7.4% to −9.4% (18% improvement) in both arms equally. More than 50% of patients, initially with very severe systolic dysfunction (GLS > −8%), were reclassified to severe (GLS −8% to −12%). LVEF did not improve in any of the groups. The quality of life measured by MLHFQ and walking distance by 6-MWT increased. Positive correlations between GLS and 6MWT (*r* = 0.41, *p* = 0.02) and GLS and MHFLQ (*r* = 0.42, *p* = 0.03) were found. The S/V + S subgroup demonstrated improvements in LVEDV (Δ16.7 vs. 4.5 ml), E/e ratio (Δ 2.8 vs. 1.4), and LAVI (Δ 9.4 vs. 8.4 ml/m^2^) as compared to ACEI + S.

**Conclusion:**

GLS, unlike LVEF, detects early changes in LV systolic function after 6–8 weeks of combined therapy, i.e., SV + S and ACE + S. GLS is more useful than LVEF in assessing early response to treatment. The effect of S/V + S and ACEI + S on LV systolic function was comparable, but the improvement in diastolic function as expressed by E/e’, LAVI, and LVEDV was more pronounced with S/V + S.

## Background

Spironolactone, ACEI, and S/V played a key role in reducing mortality and hospitalizations in patients with heart failure (HF) with reduced ejection fraction (HFrEF) (1–3). The combined therapy of ACEI + MRA or S/V + MRA fully inhibits the renin-angiotensin-aldosterone system (RAAS). They regulate fluid balance and blood pressure, ultimately modifying cardiac preload and afterload and therefore lead to beneficial changes in the structure and function of the heart, called cardiac reverse remodeling (CRR). In addition to beta-blockers, they are considered to be the most powerful drugs that initiate reverse remodeling (4).

Despite the favorable evidence for each of these drugs individually, few studies evaluate the effect of different combinations of RAAS inhibitors on CRR (4, 5). Moreover, the individual effect of either S/V or spironolactone on CRR was usually analyzed after several months, based mainly on changes in left ventricular volumes and ejection fraction (LVEF) (6, 7). Global longitudinal strain (GLS) is a well-established parameter assessing cardiac systolic function (8). A new classification of left ventricular systolic dysfunction based on GLS has been proposed (8). Only a few S/V studies have used GLS to assess CRR during therapy (8, 9).

## Aim

We aimed to compare the effects of two combined therapies of agents modifying RAAS, i.e., SV + S and ACEI + S, on L-CRR parameters. The second goal was to assess the usefulness of GLS and LVEF as indicators of CRR after 6–8 weeks of modification of heart failure therapy.

## Methods

**Figure 1:**
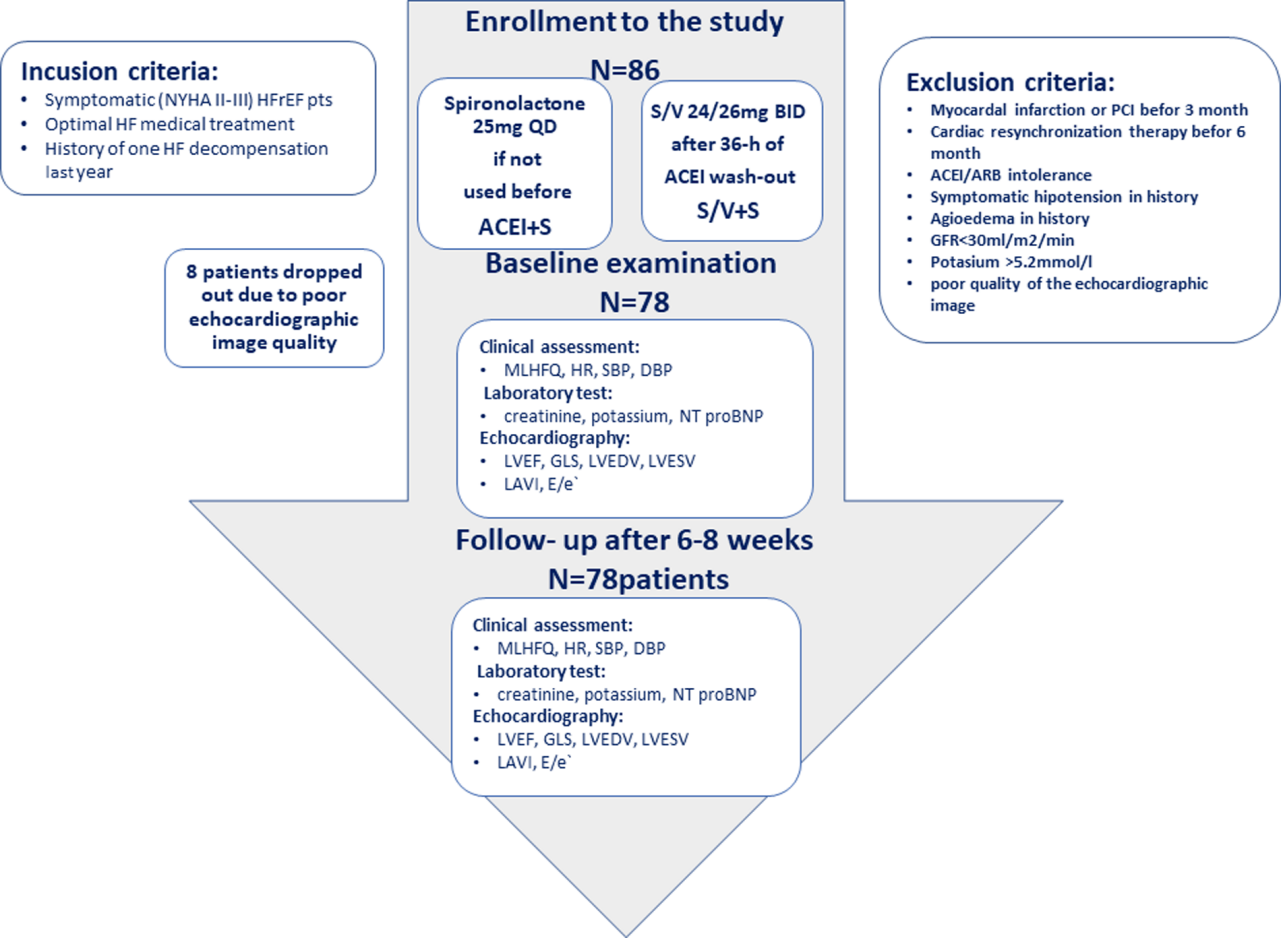
Study flow.

The study was designed as a prospective, single-center study. The study scheme is presented in Figure 1. Eighty-six patients with symptomatic heart failure with reduced ejection fraction (HFrEF), NYHA class II or III, and a history of heart failure decompensation within the past year, treated optimally according to ESC guidelines, were enrolled in the study (10). The exclusion criteria were: recent myocardial infarction, cardiac resynchronization device implantation, intolerance to angiotensin-converting-enzyme inhibitors/angiotensin receptor blockers (ACEI/ARB), previous use of S/V, symptomatic hypotension, history of angioedema, estimated glomerular filtration rate <30 ml/min/m^2^, and potassium concentration >5.2 mmol/L. At the recruitment points, all patients were on ACEIs and beta-blockers; none were on ARBs. According to the latest HF guidelines, the basic combination therapy modifying RAAS is still ACEI + MRA. If patients remain symptomatic while taking an ACEI + MRA, the ACEI should be switched to S/V (10). Our study was designed to compare the effectiveness of S/V + S therapy with ACEI + S therapy. Assignment to a given study arm was based on ongoing spironolactone therapy. The patients who had previously received spironolactone were referred to the S/V + S arm and started on S/V at a dose of 24/26 mg BID after a 36-h ACEI washout (10, 11). In the second arm of the study, consisting of patients who were MRA naive, spironolactone was initiated at 25 mg OD and ACEI was continued at the same dose. Spironolactone but not eplerenone was chosen because some data did not show a CRR benefit from eplerenone treatment (11). All individuals underwent a clinical and echocardiographic assessment at baseline and after a 1-month follow-up. The walking distance was measured by a standard 6MWT protocol (12). We used The Minnesota Living with Heart Failure (MLHFQ) to assess the quality of life (13). An echocardiographic examination was performed using a Vivid 9 Digital Ultrasound System (GE Medical Systems, United States). GLS was obtained using the semi-automatic functional imaging (AFI) method, and according to the results, the patients were assigned to the appropriate group of systolic dysfunction: very severe >−8%, severe -(8–12)%, decreased -(12–16)%, border -(16–18)%, normal -(18–20)%, supranormal <−20% (8). Information about the implemented treatment was blinded to the investigator conducting the echocardiographic examination.

The study protocol and informed consent complied with the Helsinki Convention and were approved by the Bioethical Committee of Poznan University of Medical Sciences.

### Statistical analysis

The distribution of the majority of continuous data was not normal by the Shapiro-Wilk test. Results are presented as medians with standard deviation (SD). Data were compared by the Wilcoxon test. A *p*-value <0.05 was considered statistically significant. Spearman's rank correlation coefficient was used to assess the relationship between the variables. Dell Statistica was used for the analysis (Data Analysis Software System; version 13, Dell Inc., 2016).

## Results

Eight patients were excluded due to suboptimal echocardiographic imaging. The characteristics of both subgroups, i.e., S/V + S (39 patients) and ACEI + S (39 patients), were comparable, which allowed the creation of a homogeneous group of 78 patients (mean age 63.4 SD 11.5 years, twenty females) for further analysis. The results are presented in Table 1. Fifty patients (64%) had ischemic cardiomyopathy, 57 presented NYHA class II and 21 NYHA class III symptoms. Eighteen patients (23%) had persistent atrial fibrillation (AF), 22 (28%) had stage 3 or above of chronic kidney disease, 35 (44%) had diabetes mellitus, and 8 (10%) had a history of CRT implantation. At the recruitment point, all patients were normotensive and had optimal heart rate. The baseline median NT-pro BNP value was 3,280 pg/ml. Defined daily doses of beta-blockers and loop diuretics at baseline were comparable in both subgroups (S/V + S vs. ACEI + S). All patients at baseline had LVEF ≤40%, and the median LVEF was 31.1% (31% I SV + S, 33% in ACEI + S). Forty-three patients (55%) had very severe left ventricle systolic dysfunction assessed as GLS > −8%, 24 severe as GLS −8 to −12%, and 11 decreased as GLS −16 to −18% (8). The median values of the left cardiac chamber were: LVEDV 174 ml, LVESV 126 ml, and LAVI 59 ml/m^2^. All the study results are presented in Tables 2, 3. No changes in diuretic therapy or dose increases of beta-blocker were needed in this short-time follow-up. The results were analyzed for two subgroups, i.e., S/V + S and ACEI + S, separately and for the combined group of 78 patients. During the study, renal function did not deteriorate in either the S/V + S group or the ACEI + S group (*p* = 0.3 for both groups). No reduction in the number of patients with AF was observed in the S/V + S and ACEI + S groups. Of note, no AF episode occurred during the study. After 6–8 weeks of follow-up, a significant reduction in systolic blood pressure was found, but the results were still in the optimal range (127 vs. 123 mmHg). Heart rate decreased significantly from 75 to 71 beats per minute, a trend manifested mostly in the S/V + S group. Functional capacity measured by MLHFQ improved by 7 points, and a walking distance in 6-MWT improved by 66 m. Both groups benefited equally under SV + S and ACE + S therapy. GLS changed from −7.7% to −9.4%, an 18% absolute improvement. Of note, 29 patients, initially classified as having very severe systolic dysfunction (GLS > −8%), had an improvement in GLS and were reclassified to severe systolic dysfunction (GLS −8% to −12%). GLS results are presented in Table 3. The analysis of both arms, SV + S and ACEI + S, showed a similar trend toward improvement in GLS values (*p* < 0.001). Unlike GLS, LVEF did not change in either the SV + S group or the ACE + S group (*p* = 0.9 and *p* = 0.05, respectively). Diastolic parameter E/e’ improved during SV + S therapy (18.4 vs. 15.6, *p* < 0.001) which was not observed in the ACEI + S arm (16.7 vs. 16.3, *p* = 0.06). In the SV + S group, 6–8 weeks of therapy was sufficient to reduce the LV and LA volumes, i.e., LVEDV (188 vs. 172 ml, *p* < 0.001), LAVI (67 vs. 55 ml/m^2^, *p* < 0.001). These results were not achieved in the ACEI + S group (LVEDV *p* = 0.05, LAVI *p* = 0.06).

**Table 1:** Patients characteristics at baseline (*n *= 78).

	SV + S (SD)	ACEI + S (SD)	*p* value SV + S vs. ACEI + S
No. of patients	39	39	
**Gender**			
Male (No.)	32	28	
Female (No.)	9	11	
Mean age (years)	64.3 (11.5)	62.4 (11.5)	0.06
**Aetiology**			
Non-ischemic (No.)	15	13	0.07
Ischemic (No.)	24	26	0.99
Diabetes (No.)	16	19	0.07
**Chronic kidney disease**			
Stage >3 (No.)	12	10	0.59
Atrial fibrillation (No.)	8	10	0.18
NYHA class (No.)			
I–II	28	29	0.17
III–IV	11	10	0.19
CRT (No.)	8	10	0.99
**Treatment daily dose (mg)**			
Bisoprolol	5.9 (2.7) o.d.	5.4 (3.3) o.d.	0.33
Metoprolol	43.6 (14.7) o.d.	44.8 (12.3) o.d.	0.74
Carvedilol	10.5 (4.5) b.i.d.	11.4 (6.7) b.i.d.	0.81
Furosemid	44.4 (21.3) o.d.	54.6 (26.8) o.d.	0.41
Torasemide	23.5 (8.4) o.d.	25,6 (9.7) o.d.	0.34
Sprinolactone	21. 5 (5.2) o.d.	25 (0.0) o.d.	0.68
Enalapril		5.8 (3.4) b.i.d.	
Perindopril		4.4 (1.8) o.d.	
Ramipril		6.8 (1.2) o.d.	
Sacubitril/Valsartan	24/26 b.i.d.		

ACEI, angiotensin-converting enzyme inhibitors; b.i.d., twice daily; CRT, cardiac resynchronization therapy; GFR, glomerular filtration rate; NYHA, New York Heart Association Classification; o.d., once daily; S, spironolactone; S/V, sacubitril/valsartan; SD, standard deviation.

**Table 2:** Changes in parameters during the study (*n* = 78).

Parameter	Baseline median (SD)	Follow-up median (SD)	Paired differences	
			Median	*p*-value
SBP (mmHg)	127 (14)	123 (13)	3	0.03
S/V + S	126 (12)	122 (14)	4	0.049
ACEI + S	127 (16)	126 (12)	2	0.06
DBP (mmHg)	79 (12)	77 (11)	1	0.2
S/V + S	77 (12)	77 (9)	1	0.4
ACEI + S	79 (12)	76 (10)	1	0.1
Heart rate (b.p.m.)	75 (12)	71 (10)	3	0.01
S/V + S	75 (13)	70 (10)	3	0.03
ACEI + S	78 (15)	77 (12)	0.2	0.8
MHFLQ (score)	22 (9)	16 (7)	7	<0.001
S/V + S	29 (10)	16 (8)	8	<0.001
ACEI + S	23 (9)	17 (11)	10	0.001
6MTW (m)	352 (118)	401 (108)	66	<0.001
S/V + S	392 (145)	435 (155)	72	<0.001
ACEI + S	349 (128)	368 (127)	38	<0.001
NTproBNP (pg/ml)	3,280 (4,022)	2,416 (2,678)	658	<0.001
S/V + S	3,245 (2,413)	2,414 (2,145)	671	0.01
ACEI + S	3,272 (3,922)	2,397 (2,622)	1,119	0.01
Creatinine (mg/dl)	1.2 (0.3)	1.2 (0.3)	0.1	0.3
S/V + S	1.3 (0.4)	1.2 (0.3)	0.1	0.3
ACEI + S	1.0 (0.3)	1.1 (0.3)	0.1	0.3
Potassium (mmol/L)	4.5 (0.4)	4.5 (0.4)	0.1	0.3
S/V + S	4.4 (0.4)	4.4 (0.4)	0.1	0.9
ACEI + S	4.5 (0.4)	4.6 (0.5)	0.1	0.2
LVEF (%)	31.1 (5.3)	31.5 (5.4)	0.9	0.06
S/V + S	31 (5.4)	30.6 (4.2)	0.3	0.9
ACEI + S	33 (5.2)	34 (4.2)	0.8	0.05
GLS (%)	−7.7 (2.4)	−9.4 (2.0)	1.4	<0.001
S/V + S	−8.5 (1.6)	−10.6 (2.3)	2.3	0.001
ACEI + S	−7.3 (2.3)	−8.9 (2.3)	1.5	<0.001
LVEDV (ml)	174 (50)	163 (45)	4.6	<0.001
S/V + S	188 (51)	172 (46)	4.2	<0.001
ACEI + S	170 (24)	169 (22)	2.5	0.05
LVESV (ml)	126 (44)	117 (24)	3.6	<0.001
S/V + S	134 (44)	122 (40)	3.8	0.001
ACEI + S	128 (21)	125 (47)	1.8	0.03
LAVI (ml/m^2^)	59 (22)	51 (26)	5.4	0.001
S/V + S	67 (21)	55 (21)	7.5	<0.001
ACEI + S	55 (12)	53 (9)	1.8	0.06
E/e’	16.8 (6.7)	16.0 (5.2)	1.3	<0.001
S/V + S	18.4 (7.0)	15.6 (5.9)	2.8	<0.001
ACEI + S	16.7 (4.0)	16.3 (3.4)	0.9	0.06

ACEI, angiotensin-converting enzyme inhibitors; DBP, diastolic blood pressure; GLS, global longitudinal strain; LAVI, left atrial volume index; LVEDV, left ventricle end-diastolic volume; LVEF, left ventricle ejection fraction; LVESV, left ventricle end-systolic volume; MLHFQ, Minnesota Living with Heart Failure Questionnaire; SD, standard deviation; SBP, systolic blood pressure; S, spironolactone; S/V, sacubitril/valsartan; 6MWT, 6-minute walk test.

**Table 3:** Number of patients whose GLS and LVEF changed during the study.

LV systolic function according to GLS (−%) and LVEF (%)	No. of patients at the baseline	No. of patients at the follow-up
Very severe: GLS > −8	43	14
SV + S	20	9
ACEI + S	13	5
Severe: GLS—(8–12)	24	53
SV + S	13	33
ACEI + S	11	20
Decreased: GLS—(12–16)	11	11
SV + S	6	7
ACEI + S	5	4
Border: GLS—(16–18)	0	0
Normal and supranormal: GLS—< −18	0	0
LVEF < 40%	78	71
SV + S	39	36
ACEI + S	39	35
LVEF > 40%	0	7
SV + S	0	3
ACEI + S	0	4

ACEI, angiotensin-converting enzyme inhibitors; GLS, global longitudinal strain; LVEF, left ventricle ejection fraction; LVEF, left ventricle ejection fraction.

## Discussion

We have proved that GLS is a sensitive parameter for early assessment of the effects of pharmacological treatment in symptomatic patients with a heart failure with reduced ejection fraction. The 18% absolute improvement in GLS after 6–8 weeks of therapy modification likely results from favorable hemodynamic changes. According to other authors, decreasing serum NT-proBNP levels can be detected as early as after 1 month of S/V therapy (14, 15) and after 4 months of spironolactone therapy (7). Recent studies show that these changes correlate with a reduction in the indexed left atrial volume (LAVI) and E/e’ ratio, reflecting cardiac filling pressures (15). Moreover, a decrease in the heart rate, particularly noticeable in the S/V + S subgroup (median drop −3 per minute), could prolong the diastolic phase and thus improve left ventricular contractility (6). This phenomenon was independent of either beta-blocker or ivabradine dosage escalation. In contrast to previous studies, LVEF did not improve in our study (baseline vs. follow-up: 30.1 vs. 30.5%). However, it should be noted that the observation period in these studies was at least 3 months or longer, and S/V was mostly titrated to recommended dose (6, 14, 16). However, it is not surprising that the following L-CRR parameters improved: LVESV, LVEDV, LVEF, LAVI. Our study focused on how a low dose of S/V or spironolactone affects L-CRR in HFrEF patients. In the real world, in contrast to clinical trials, prescription and up-titration of GMDT remains low mostly due to clinical inertia or complex patients’ characteristics (17, 18). There is paucity of data on how effective S/V or spironolactone are in low, suboptimal doses in patients for whom the dose cannot be increased for various reasons. We hypothesize that the lack of LVEF improvement in our study can be explained by the greater dependency of LVEF on mid-wall circumferential fibers function (19). Thus, to improve the longitudinal and radial strain, a longer treatment time is needed. Further research is warranted to explore this topic. The noticeable improvement in GLS compared to LVEF obtained in a relatively short time after adding a low dose of S/V or spironolactone to the basic therapy convinces us that GLS may be a better tool for monitoring therapy progress.

We observed an improvement in quality of life. MLHFQ scores decreased by an average of −7 points. These results align with the PARADIGM-HF study (1) and are relevant, as quality of life is an important heart failure treatment goal.

The distance in 6-MWT increased by an average of 66 m in both treatment arms, without significant differences between them. According to some authors “minimal important difference” is 35 meters (12). Significant improvement in exercise capacity measured by 6-MWT was appreciable in both groups, i.e., S/V + S and ACEI + S (median difference 72 vs. 38 m, *p* < 0.001, respectively). Recent studies revealed similar results. In OUTSTEP- HF study, after 12 weeks on S/V, 6MWT improved by 35.09 m, and the benefit is comparable to the enalapril group by 26.11 m (20). In the spironolactone study, improvement in exercise tolerance measured by 6-MWT was dose-dependent, but significant in both low and high doses (21). The 6-MWT is an independent predictor of all-cause mortality in HF; thus, our results may be related to a better prognosis (22). Spironolactone, SV, and enalapril are all RAA modifiers. The mechanisms behind improvements in exercise capacity and quality of life during RAAS inhibitors therapy are a subject of debate. The correlations between GLS and 6MWT (*r* = 0.41, *p* = 0.02) and GLS and MHFLQ (*r* = 0.62, *p* = 0.03) may advocate influence on systolic function. Other hypotheses include lowering filling pressures and peripheral vasodilation (23). The differences in the results between S/V + S and ACEI + S therapy are worth noting. LVEDV, LAVI, and E/e’ improved significantly in S/V + S arm. Our results are consistent with the outcomes of the EVALUATE-HF study, which showed a significant reduction in LAVI and E/e’ under the S/V, in contrast to enalapril (24). According to recent data, LA function is not only a consequence of increased left ventricular filling pressure but has been proposed as a clinically significant separate entity (25). A new parameter known as left atrial function index (LAFI), which depends on left atrial emptying fraction (LAEF), LVEF, and LAVI, was proposed to assess LA function. The deterioration of LAFI corresponds to the worsening of symptoms of heart failure, deterioration of quality of life and life expectancy in patients with HFrEF (26, 27). Patients with complete L-CRR of both the LA and LV are significantly less likely to experience HF exacerbations or death compared to patients with incomplete remodeling (28). Of note, the change in LAVI was likely not a consequence of arrhythmias because the proportion of patients with atrial fibrillation did not change during the observation period. Thus, we believe S/V + S therapy appears to be more effective in inducing CRR than ACEI + S. Further studies are required to assess these findings.

## Conclusion

GLS, unlike LVEF, demonstrated early, modest changes in left ventricular systolic function after 6–8 weeks in both groups of combined RAAS inhibitors therapy, i.e., SV + S and ACE + S. The correlation between GLS and clinical improvement suggests that GLS may be more useful than LVEF in assessing early clinical response to treatment.

The effect of S/V + S and ACEI + S on LV systolic function was comparable, but the improvement in LV diastolic function as assessed by E/e’, LAVI and LVEDV was more pronounced with S/V + S therapy after 6–8 weeks of treatment.

Short-term observation of combined therapies of S/V + S and ACEI + S, did not reveal any particular side effects.

## Limitations

The main limitations of our prospective study is a small group of participants derived from single center, and a relatively short-term observation period. Deficit of older patients and the predominance of men, mainly due to ischemic etiology, are also major limitations. Little racial diversity should also be pointed out. Therefore, the results of our study should be applied to the general HFrEF population with caution. Further studies conducted on a larger group of HFrEF patients with multimorbidity's are required.

## Data Availability

The data analyzed in this study is subject to the following licenses/restrictions: The data that support the findings of this study are available on request from the corresponding author. The data are not publicly available due to privacy or ethical restrictions. Requests to access these datasets should be directed to wioletta.sacharczuk@wp.pl.
